# Microbial communities in top- and subsoil of repacked soil columns respond differently to amendments but their diversity is negatively correlated with plant productivity

**DOI:** 10.1038/s41598-019-45368-9

**Published:** 2019-06-20

**Authors:** Corinne Celestina, Jennifer L. Wood, James B. Manson, Xiaojuan Wang, Peter W. G. Sale, Caixian Tang, Ashley E. Franks

**Affiliations:** 1 Department of Physiology, Anatomy and Microbiology, La Trobe University, Bundoora, VIC 3086 Australia; 2 Centre for Future Landscapes, La Trobe University, Bundoora, VIC 3086 Australia; 3 Department of Animal, Plant and Soil Sciences, AgriBio the Centre for AgriBiosciences, La Trobe University, Bundoora, VIC 3086 Australia

**Keywords:** Soil microbiology, Microbial ecology

## Abstract

Organic and inorganic amendments with equivalent nutrient content may have comparable fertilizer effects on crop yield, but their effects on the soil microbial community and subsequent plant-soil-microbe interactions in this context are unknown. This experiment aimed to understand the relationship between soil microbial communities, soil physicochemical characteristics and crop performance after addition of amendments to soil. Poultry litter and synthetic fertilizer with balanced total nitrogen (N) content equivalent to 1,200 kg ha^−1^ were added to the topsoil (0–10 cm) or subsoil layer (20–30 cm) of repacked soil columns. Wheat plants were grown until maturity. Soil samples were taken at Zadoks 87–91 (76 days after sowing) for analysis of bacterial and fungal communities using 16S and ITS amplicon sequencing. The interaction between amendment type and placement depth had significant effects on bacterial and fungal community structure and diversity in the two soil layers. Addition of poultry litter and fertilizer stimulated or suppressed different taxa in the topsoil and subsoil leading to divergence of these layers from the untreated control. Both amendments reduced microbial community richness, diversity and evenness in the topsoil and subsoil compared to the nil-amendment control, with these reductions in diversity being consistently negatively correlated with plant biomass (root and shoot weight, root length, grain weight) and soil fertility (soil NH_4_^+^, shoot N). These results indicate that in this experimental system, the soil microbial diversity was correlated negatively with plant productivity.

## Introduction

Surface^1^ and subsoil^2–4^ placement of nutrient-rich organic amendments has been used as a technique to improve plant productivity on agricultural soils characterized by low organic matter, poor physical structure or low chemical fertility. Crop yield responses to the amendments have been attributed to a combination of improved soil structure, water use, nutrient supply and biological activity^2–6^. Because of the interplay between the plant, soil and soil biota^7^, it is expected that the microbial community plays a key role in nutrient transformations and soil aggregation and hence the crop response to organic amendments. Although many authors have reported changes to soil properties and microbial communities^8–11^ after addition of organic amendments to soil, our understanding of the subsequent plant-soil-microbe interactions and their effect on plant productivity is unclear.

Additionally, the role of nutrients in the crop response to organic amendments may be more significant than previously thought, with a number of recent meta-analyses concluding that organic amendments do not have substantial additional effects on crop yields beyond their fertilizer effects^12–15^. That is, organic and inorganic amendments with matched macronutrients can have the same effect on crop yields, regardless of whether they are applied to the soil surface or the subsoil^2,3^. This suggests that the key contribution of the microbial community to the crop response to amendments may lie in its role in nutrient cycling and decomposition. And, whilst plant productivity may frequently be nutrient-limited, soil microorganisms may be limited by other factors and so their responses to amendments may differ^16^.

In the present study we aimed to understand the relationship between soil microbial communities, soil physicochemical characteristics and crop performance after addition of organic and inorganic amendments to repacked soil columns. Poultry litter (‘PL’) and synthetic fertilizer (‘FERT’) with balanced total nitrogen (N) content were added to topsoil (‘TOP’) or subsoil (‘SUB’) layers of soil columns with wheat plants grown until physiological maturity. We hypothesized that the structure and function of the microbial community would differ between different amendments and different soil layers, with the addition of amendments leading to convergence of the bacterial and fungal communities of the topsoil and subsoil layers. Furthermore, we theorized that changes in microbial community structure and diversity compared to the nil-amendment control (‘NIL’) would be positively associated with soil fertility, aggregation and plant growth.

## Results

A total of 1,953,805 and 1,079,219 high quality 16S and ITS sequences were obtained from the 18 samples, with sequences per sample ranging from 34,714 to 193,000 for bacteria and 26,744 to 117,578 for fungi. These sequences were clustered into 6,689 bacterial and 570 fungal OTUs at the 97% similarity level with an average of 1,810 bacterial and 150 fungal OTUs per sample. After trimming to remove OTUs with mean relative abundance less than 0.005% there were 538 bacterial and 110 fungal OTUs remaining. The majority of OTUs were rare members of the community, with only 2% of bacterial and 10% of fungal OTUs having mean relative abundance > 1% (Supplementary Table S1a, 1b).

### Comparison of microbial community structure

The estimated number of OTUs in each sample (Chao1 richness), Shannon diversity and Simpson’s evenness are shown in Table 1. The type of amendment and placement depth had consistent, significant (*P* < 0.05) effects on the bacterial and fungal communities, but the amendment × depth interactive effect was not as strong. Additionally, placement depth tended to have a stronger effect size than amendment type or amendment × depth interaction. In general, addition of fertilizer and, to a lesser extent, poultry litter to the soil reduced the richness, diversity and evenness of bacterial and fungal communities in both soil layers. As a main effect, averaged across placement depth, the nil-amendment control tended to have the highest bacterial and fungal richness, diversity and evenness, and the fertilizer treatment tended to have the lowest values for all indices. These reductions in Chao1 richness and Shannon diversity are due to reductions in the number of OTUs and the distribution of individuals among OTUs^17–19^, whereas reductions in evenness correspond to a less evenly distributed community dominated by a few OTUs^19^. In terms of placement depth, microbial communities in the subsoil consistently had lower richness, diversity and evenness compared to those in the topsoil. The exception to the trend was Simpson’s evenness in fungal communities: evenness was not affected by amendment type but did differ between topsoil and subsoil layers, with the subsoil significantly more even (*P* = 0.022) because of more equal abundances of all OTUs.

**Table 1:** Richness, diversity and evenness of microbial communities in the six amendment × depth treatments. HSD, Tukey’s honest significant difference; n.s., not significant at *P* < 0.05.

	Bacteria			Fungi		
	Chao1 richness	Shannon diversity (*H*)	Simpson’s evenness (*E*)	Chao1 richness	Shannon diversity (*H*)	Simpson’s evenness (*E*)
**Treatment**						
Topsoil + Nil	484	5.32	0.231	76	2.24	0.050
Topsoil + Poultry litter	494	5.37	0.247	70	1.50	0.031
Topsoil + Fertilizer	463	5.02	0.194	66	0.97	0.024
Subsoil + Nil	481	5.28	0.230	63	2.08	0.064
Subsoil + Poultry litter	450	4.92	0.144	41	1.70	0.095
Subsoil + Fertilizer	480	4.43	0.037	40	0.96	0.061
**Amendment type**						
Nil	483	5.30	0.23	69	2.16	0.057
Poultry litter	472	2.14	0.195	55	1.60	0.063
Fertilizer	471	4.72	0.116	53	0.97	0.042
**Placement depth**						
Topsoil	481	5.23	0.224	70	1.58	0.034
Subsoil	470	4.88	0.137	48	1.57	0.073
**HSD (** ***P*** ** = 0.05)**						
Amendment type	n.s.	0.24	0.048	n.s.	0.39	n.s.
Placement depth	n.s.	0.16	0.032	14	n.s.	0.022
Amendment type × Placement depth	46	0.43	0.085	n.s.	n.s.	n.s.

**Figure 1:**
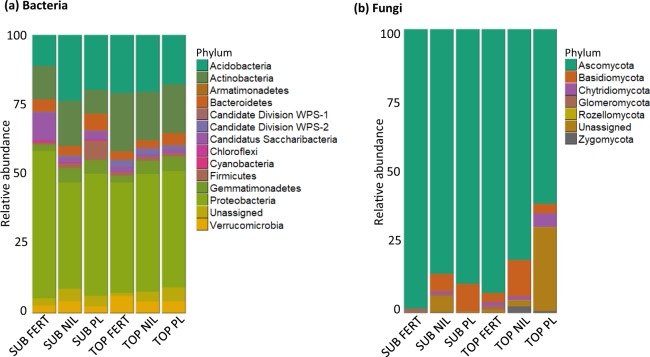
Relative abundance of bacterial **(a)** and fungal **(b)** phyla in microbial communities in topsoil (TOP) or subsoil (SUB) treated with chemical fertilizer (FERT), poultry litter (PL) or no amendment (NIL).

A similar phylum-level composition was observed across all six treatments (Fig. 1). The bacterial community in the topsoil and subsoil was dominated by Proteobacteria (~45%), Acidobacteria (~19%) and Actinobacteria (~16%), whilst Ascomycota (~85%) was the dominant fungal phylum in both soil layers. Fungal community composition was variable between placement depth and amendment type, whereas the bacterial community was relatively stable in the topsoil but more variable in the subsoil across the three amendment treatments. In the nil-amendment control, the topsoil and the subsoil had similar phylum-level abundances, but differences could be seen in community composition between the two soil layers in the fertilizer and poultry litter treatments.

**Figure 2:**
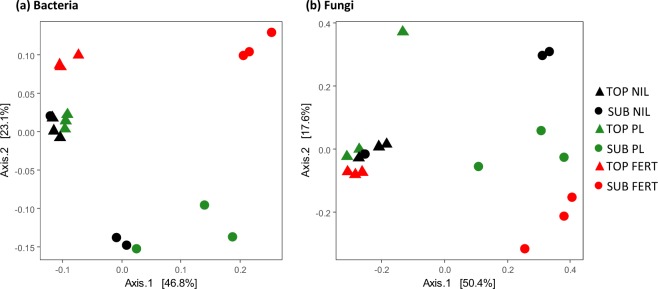
Ordination of principal coordinates analysis of weighted UniFrac distances between bacterial (**a**) and fungal (**b**) communities in topsoil (TOP) or subsoil (SUB) treated with chemical fertilizer (FERT), poultry litter (PL) or no amendment control (NIL).

Principal coordinates analysis of weighted UniFrac distances was used to visualize the effects of amendment type and placement depth on soil microbial communities (Fig. 2). The first two principal coordinates explained a high percentage of the variance for the bacterial (70%) and fungal communities (68%), with variation in microbial community structure associated with both placement depth and amendment type. Within each depth, communities in the nil-amendment control, fertilizer and poultry litter treatments separated. There was also clear separation between the microbial communities of the topsoil and subsoil, although one replicate of the nil-amendment subsoil treatment clustered with the nil-amendment topsoil. Overall, the topsoil communities clustered more closely together and were more similar in terms of species abundance and phylogenetic distance than the subsoil communities.

Permutational multivariate analysis of variance (PERMANOVA) of weighted UniFrac distances confirmed a significant (*P* < 0.05) global effect of amendment type, placement depth and amendment type × placement depth on microbial communities (Table 2). As a main effect, placement depth (Pseudo-*F* = 13.79–14.31) had a stronger effect than amendment type (Pseudo-*F* = 2.66–6.20) and effect sizes were generally higher in bacteria than in fungi. There were significant differences in microbial communities between nil-amendment and fertilizer treatments, and poultry litter and fertilizer treatments. Bacterial communities also differed between nil-amendment and poultry litter treatments.

**Table 2:** Results of permutational analysis of variance (PERMANOVA) of weighted UniFrac distances testing the effect of amendment type and depth on soil microbial communities. *P*(MC) = *P*-value based on Monte Carlo random draws.

	Bacteria		Fungi	
	Pseudo-F	*P* -value	Pseudo-F	*P* -value
**Global tests**				
Amendment type	6.20	**0.001**	2.66	**0.003**
Placement depth	14.31	**0.002**	13.79	**0.001**
Amendment type × Placement depth	3.65	**0.002**	2.46	**0.006**
**Pairwise tests**				
Amendment type	**t-statistic**	***P*** **-value**	**t-statistic**	***P*** **-value**
Nil vs. Fertilizer	3.42	**0.002**	2.33	**0.006**
Nil vs. Poultry Litter	1.47	**0.048**	1.14	0.278
Poultry Litter vs. Fertilizer	2.61	**0.003**	1.69	**0.013**
Amendment type within Placement depth	**t-statistic**	***P*** **(MC)**	**t-statistic**	***P*** **(MC)**
Topsoil				
Nil vs. Fertilizer	2.76	**0.012**	1.78	0.078
Nil vs. Poultry Litter	1.33	0.199	1.12	0.323
Poultry Litter vs. Fertilizer	4.51	**0.002**	1.30	0.253
Subsoil				
Nil vs. Fertilizer	3.12	**0.007**	2.25	**0.036**
Nil vs. Poultry Litter	1.34	0.213	1.25	0.254
Poultry Litter vs. Fertilizer	2.04	**0.037**	2.02	0.071
Placement depth within Amendment type	**t-statistic**	***P*** **(MC)**	**t-statistic**	***P*** **(MC)**
Nil				
Topsoil vs. Subsoil	1.49	0.173	1.69	0.141
Poultry litter				
Topsoil vs. Subsoil	2.25	**0.032**	2.00	**0.036**
Fertilizer				
Topsoil vs. Subsoil	4.50	**0.001**	8.48	**0.004**

Pairwise PERMANOVA revealed a significant interactive effect of amendment type and placement depth. Comparing the two placement depths of each amendment, poultry litter and fertilizer both showed differences (*P*(MC) < 0.05) in bacterial and fungal community structures between the topsoil and the subsoil. However, the difference between microbial communities in the nil-amendment topsoil and subsoil was not significant (*P*(MC) > 0.05) due to one replicate from the subsoil layer that clustered with the topsoil samples (Fig. 2). Comparing between amendment types within each placement depth, the bacterial community in the synthetic fertilizer treatment was significantly different to both the nil-amendment and poultry litter treatments in the topsoil and subsoil. This trend was similar for fungal communities in the subsoil, with a significant difference (*P*(MC) = 0.036) observed between nil and fertilizer treatments and some indication of dissimilarity between poultry litter and fertilizer (pseudo-*F* = 2.02, *P*(MC) = 0.071). Amongst fungal communities in the topsoil, there was no significant difference between amendments. However, this was only the case for PERMANOVA of weighted UniFrac distances (based on phylogenetic structure, weighted by OTU abundance). The PERMANOVA of unweighted UniFrac distances (takes into account species presence only), suggests that although similar OTUs were present, the abundance of these OTUs was likely having a strong influence on community structure in this layer (Poultry litter vs. Fertilizer, *P*(MC) = 0.04; Nil vs. Fertilizer, *P*(MC) = 0.04) (Supplementary Table S2).

### Comparison of microbial communities at OTU level

**Figure 3:**
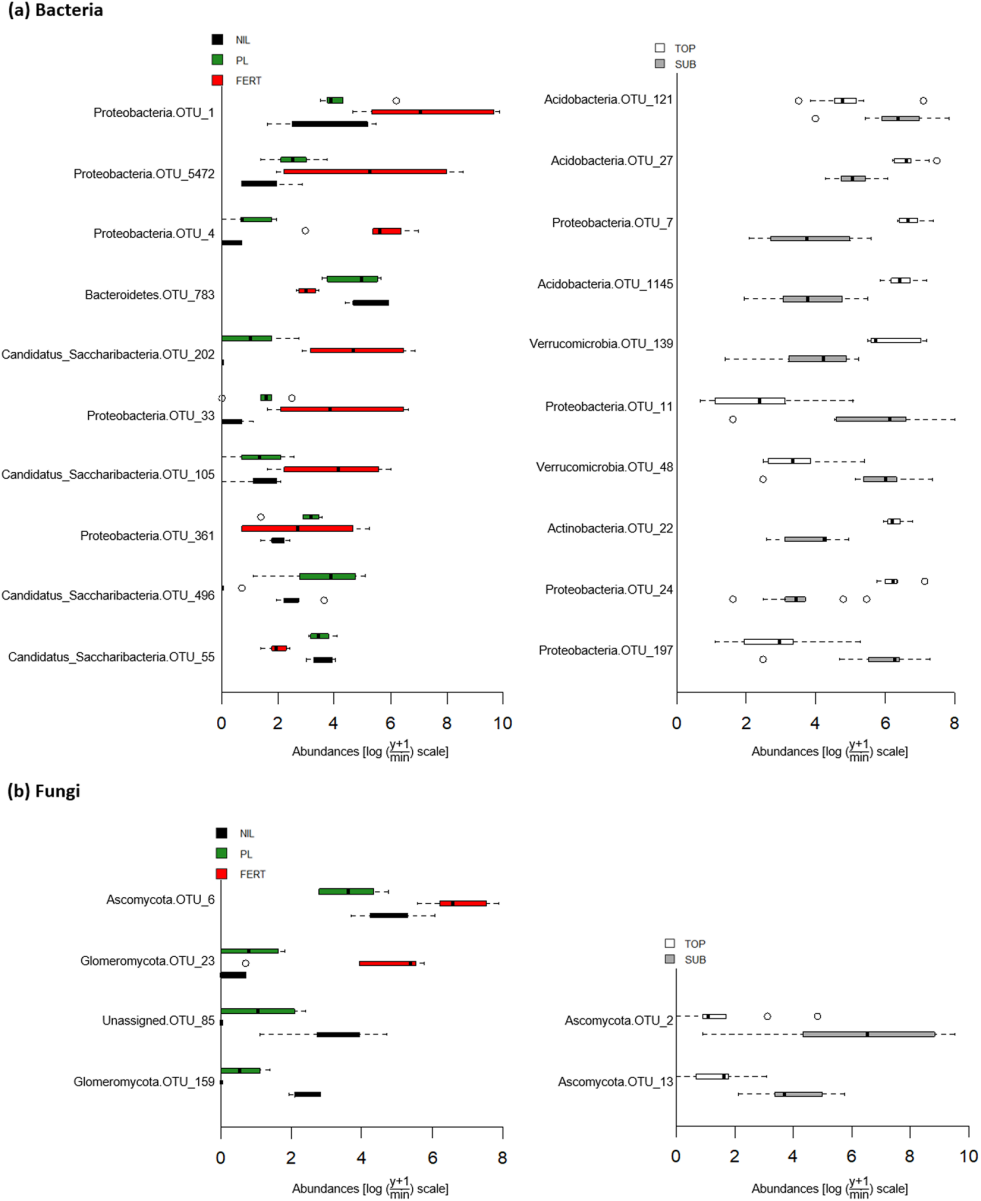
Abundances of bacterial (**a**) and fungal (**b**) OTUs that significantly (*P*_adj_ < 0.05) differed between topsoil (TOP) and subsoil (SUB) or between soils amended with chemical fertilizer (FERT), poultry litter (PL) or no amendment control (NIL). Only the top 10 most abundant bacterial OTUs are shown.

Univariate tests for each OTU identified 99 bacterial and 6 fungal OTUs (16 and 5% of the total community, respectively) that were observed to differ significantly (*P*_adj_ < 0.0.5) between amendment type and placement depth treatments (Supplementary Table S3). The majority of bacterial OTUs (84 of 99) were responding to the placement depth, whereas only 12 out of 99 OTUs differed in abundance between the three amendments and the remainder responded to the amendment × depth interaction. Most of the bacterial OTUs that responded to amendment type belonged to the phyla Proteobacteria and Candidatus Saccharibacteria (Candidate Division TM7). Those responding to placement depth were mainly from the highly abundant phyla Proteobacteria, Acidobacteria and Actinobacteria (subset shown in Fig. 3a). Among the bacterial OTUs that differed between the amendment types, 8 out of 12 OTUs had a higher abundance in the fertilizer-amended soil than in either the nil or poultry litter treatments, and 7 OTUs were lowest in abundance in the nil treatment. Of the bacterial OTUs responding to placement depth, 55 of 84 were more abundant in the topsoil compared to the subsoil (Fig. 3a, Supplementary Table S3). Of the OTUs that were more abundant in the topsoil, the order of abundance (from highest to lowest) was most commonly TOP FERT > TOP PL > TOP NIL» SUB NIL > SUB FERT > SUB PL. Of the OTUs that were more abundant in the subsoil, the order of abundance was most commonly SUB PL > SUB NIL > SUB FERT≫ TOP NIL > TOP PL > TOP FERT (Supplementary Table S1, S4). Of the 6 fungal OTUs that were observed to differ significantly in abundance, 4 responded to the amendment and 2 to the placement depth (Fig. 3b). Half were from the phylum Ascomycota and another two were classified as Glomeromycota. There were no consistent trends in fungal OTUs responding to amendment type, but the two OTUs that responded to depth were both higher in abundance in the subsoil than the topsoil (Fig. 3b).

### Linking microbial community structure to soil and plant variables

**Figure 4:**
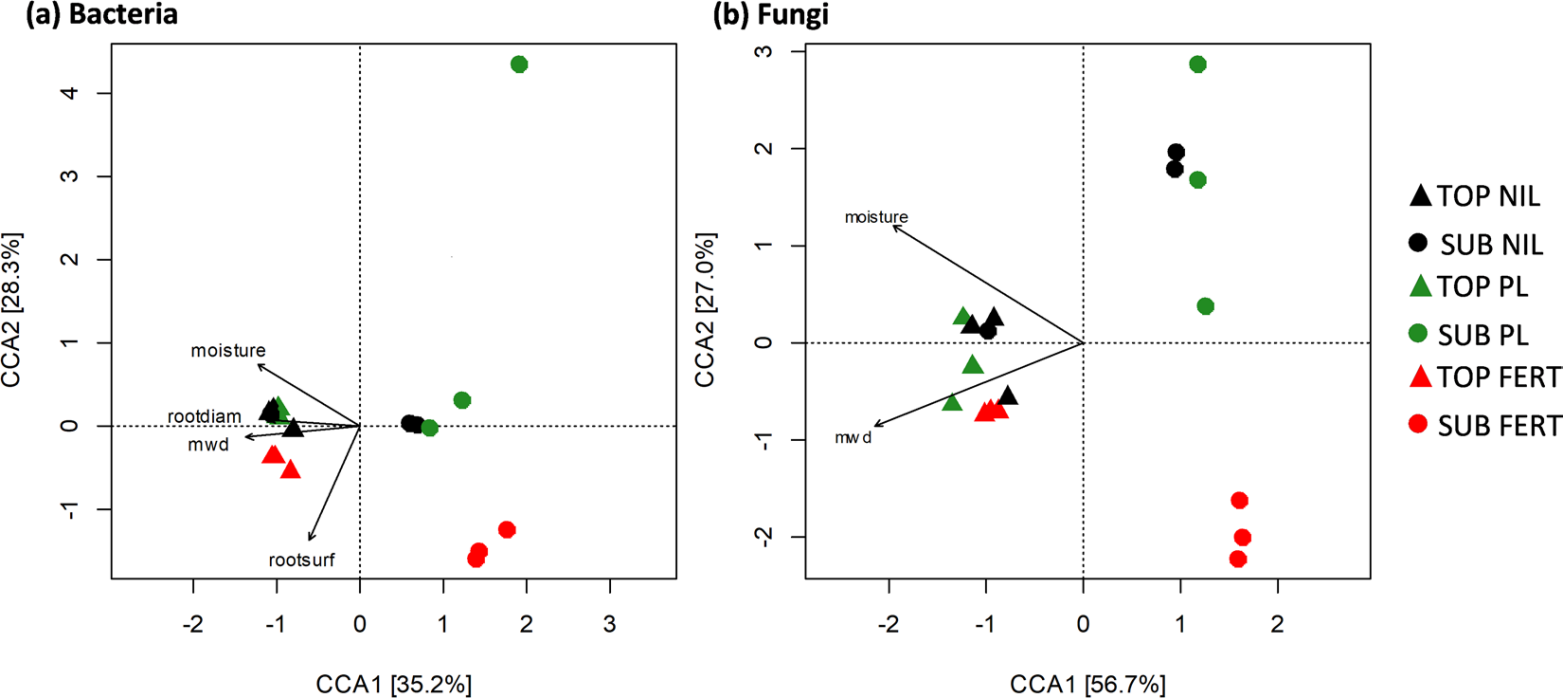
Canonical correspondence analysis (CCA) biplot of plant and soil variables that significantly influence bacterial **(a)** and fungal **(b)** communities in topsoil in topsoil (TOP) or subsoil (SUB) treated with chemical fertilizer (FERT), poultry litter (PL) or no amendment control (NIL). Variables: moisture, gravimetric moisture content; rootdiam, root diameter; mwd, aggregate mean weight diameter; root surf, root surface area.

Canonical correspondence analysis (CCA) was used to relate measured soil and plant variables to bacterial and fungal community structure. After removing highly correlated variables (soil NH_4_^+^, large macroaggregates, small macroaggregates, microaggregates, silt and clay fraction and total root weight), stepwise ordination significance testing on the remaining measures (soil NO_3_^−^, gravimetric moisture, aggregate mean weight diameter, total shoot weight, root length, root surface area, root diameter, shoot N and total grain weight) identified six environmental variables that were significantly correlated with bacterial and/or fungal community structure (Fig. 4). Soil moisture content (*P* = 0.005), mean-weight diameter of aggregates (*P* = 0.005), root surface area (*P* = 0.035) and root diameter (*P* = 0.020) had a strong influence on bacterial community structure. Soil moisture (*P* = 0.005) and aggregate mean-weight diameter (*P* = 0.025) were also strong drivers of fungal community structure. The constrained ordinations distinctly separated into topsoil and subsoil groups. All six significantly-correlated variables were higher in value in the topsoil than subsoil (Supplementary Table S4) and this corresponds with the direction of the vectors on the ordinations (Fig. 4). The CCA-based variation partitioning analysis indicated that the retained variables (moisture, mean-weight diameter of aggregates, root diameter and root surface area) explained 16, 10, 4 and 7% of the observed variance in bacterial communities, respectively. The variables significantly correlated with fungal community structure (moisture, mean-weight diameter of aggregates) explained 8 and 6% of the community structure. The majority of variation in bacterial and fungal communities was unexplained.

Alpha diversity indices (Chao1 richness estimator, Shannon diversity and Simpson’s evenness) for bacteria and fungi were frequently negatively correlated (*P* < 0.05) with environmental variables (Table 3). Reduced OTU richness, diversity and evenness were consistently associated with increasing concentrations of inorganic N in the soil and of total N in shoots, as well as increasing above- and below-ground plant biomass. There were significant (*P* < 0.05), strong negative correlations with soil ammonium, shoot N, root weight, shoot weight, root length and grain weight in both the topsoil and subsoil. Conversely, soil moisture was positively correlated (*P* < 0.05) with bacterial and fungal Shannon diversity in both soil layers. In the subsoil only, Shannon diversity and bacterial Simposon’s evenness were also found to increase with increasing proportion of silt and clay fractions. In terms of the other measures of soil structure, diversity indices tended to positively correlate with the proportion of soil aggregates and aggregate mean-weight diameter in the topsoil, but negatively correlate with those in the subsoil. Bacterial Chao1 richness and fungal community evenness was weakly correlated with most environmental variables in the subsoil.

**Table 3:** Pearson’s correlation coefficients between microbial diversity indices and environmental variables in the topsoil and subsoil. Asterisks (*) indicate significant correlations between variables at *P* < 0.05 level.

	Bacteria			Fungi		
	Chao1 richness	Shannon diversity (*H*)	Simpson’s evenness (*E*)	Chao1 richness	Shannon diversity (*H*)	Simpson’s evenness (*E*)
**Topsoil**						
Soil NH_4_^+^	−0.51	−0.77*	−0.62	0.18	−0.66	−0.53
Soil NO_3_^−^	−0.45	−0.40	−0.17	−0.12	−0.26	−0.28
Moisture	0.52	0.92*	0.93*	0.53	0.71*	0.17
Large macroaggregates	0.17	0.42	0.35	−0.42	0.46	0.45
Small macroaggregates	0.43	0.46	0.40	0.12	0.16	−0.07
Microaggregates	0.11	0.10	0.01	0.09	0.11	0.02
Silt and clay fraction	−0.36	−0.57	−0.47	0.27	−0.47	−0.33
Aggregate diameter	0.21	0.46	0.38	−0.40	0.48	0.44
Root weight	−0.75*	−0.89*	−0.65	−0.59	−0.82*	−0.52
Shoot weight	−0.60	−0.81*	−0.57	−0.52	−0.93*	−0.64
Root length	−0.71*	−0.96*	−0.79*	−0.45	−0.82*	−0.50
Root surface area	−0.05	−0.59	−0.82*	−0.24	−0.34	0.07
Root diameter	−0.57	−0.52	−0.24	−0.29	−0.34	−0.42
Shoot N	−0.76*	−0.91*	−0.67*	−0.40	−0.69*	−0.45
Grain weight	−0.46	−0.66	−0.48	−0.67*	−0.83*	−0.47
**Subsoil**						
Soil NH_4_^+^	0.11	−0.70*	−0.73*	−0.57	−0.80*	−0.04
Soil NO_3_^−^	0.16	−0.56	−0.61	−0.54	−0.67*	−0.03
Moisture	−0.25	0.77*	0.83*	0.39	0.84*	0.16
Large macroaggregates	−0.03	−0.54	−0.60	−0.63	−0.57	0.31
Small macroaggregates	0.27	−0.37	−0.30	0.13	−0.34	−0.61
Microaggregates	−0.56	−0.61	−0.56	−0.50	−0.48	0.12
Silt and clay fraction	0.09	0.80*	0.81*	0.63	0.77*	−0.02
Aggregate diameter	−0.01	−0.56	−0.63	−0.62	−0.59	0.26
Root weight	0.07	−0.75*	−0.80*	−0.50	−0.84*	−0.04
Shoot weight	0.00	−0.84*	−0.88*	−0.51	−0.88*	−0.01
Root length	−0.12	−0.88*	−0.93*	−0.58	−0.90*	0.04
Root surface area	0.75*	0.06	0.05	0.53	0.02	−0.48
Root diameter	0.12	0.02	0.16	0.37	0.05	−0.56
Shoot N	0.24	−0.87*	−0.90*	−0.34	−0.93*	−0.36
Grain weight	0.07	−0.80*	−0.87*	−0.47	−0.85*	−0.03

## Discussion

There is evidence that organic and inorganic amendments with equivalent total nutrient content have comparable fertilizer effects on crop yield^12–15^. However, the effects of these amendments on the soil microbial community, and subsequent plant-soil-microbe interactions, are unknown. This experiment aimed to understand the relationship between soil microbial communities, soil physicochemical characteristics and crop performance after addition of amendments with equivalent total N content to topsoil and subsoil.

Amendment type and placement depth affected the diversity of bacterial and fungal communities in the soil, with the depth of placement tending to have a stronger effect than the amendment type. Topsoil communities had a larger number of OTUs and were richer and more evenly distributed than subsoil communities, regardless of whether there was an amendment added to the soil or not. Nevertheless, both organic and inorganic amendments tended to reduce bacterial and fungal community richness, diversity and evenness in both soil layers compared to the nil control because they favored the growth of a few OTUs that dominated the community of the amended soil. The slow-release fertilizer had a larger effect than the poultry litter, reflecting the effect of the nutrient-rich amendments on measured soil and plant variables (e.g. soil inorganic N, shoot weight, root length), which tended to respond in the order of fertilizer > poultry litter > nil-amendment control. Perturbations like the addition of nutrients or labile carbon to a soil have frequently been shown to reduce microbial community diversity and are likely to have a strong selective effect on the community^20–22^. The highly available nutrients in the fertilizer treatment may have negatively affected microbial community structure and diversity by increasing the concentration of salts in the soil solution. The poultry litter, on the other hand, may have had a less pronounced effect than the synthetic fertilizer treatment because it was slower to decompose and the nutrients it contained were not immediately available^23^, or because at the time of sampling the effect of this amendment was no longer apparent. Surprisingly, the addition of poultry litter to the soil was also not observed to increase the alpha diversity metrics of the community, despite this manure source being known to contain large amounts of predominantly bacterial species^24^. Other authors have shown significant, rapid and long-lasting effects of manure-based organic amendments on soil microbial communities^10,25,26^, including inoculation of species into the soil from the exogenous manure sources^27^. It is not clear from the present study how quickly these amendment-mediated changes in microbial community structure and diversity arise, nor how long they persist, given the single sampling time point for microbial analysis. Additional harvest times would be necessary to understand the successional trajectories of microbial communities after the addition of different amendments.

Amendment type and placement depth also had significant effects on microbial community structure, and again, the effect of placement (topsoil vs. subsoil) was more pronounced than that of amendment type. Of the OTUs that differed in abundance in response to placement depth, the majority were higher in abundance in the topsoil than the subsoil, indicating a larger stimulatory effect in the topsoil layer. This contradicts other studies reporting a more pronounced response to amendment addition in deeper soil layers under field conditions, leading to convergence of topsoil and subsoil layers^28^. Additionally, all of the OTUs that differed in abundance were inherently more abundant in one soil layer than the other, regardless of whether the soil was amended or not. This suggests that we were primarily observing natural variation in the microbial communities that was arising due to the depth of the soil layer, rather than the type of amendment. Nevertheless, there were obvious trends in each layer caused by the amendments that indicates an interaction between placement depth and amendment type. The poultry litter and inorganic fertilizer treatments formed distinct microbial communities in the topsoil and subsoil layers. Our observations here were directly contrary to our hypothesis: the nutrient-rich amendments led to divergence instead of convergence of the microbial communities of the top- and subsoil. Thus, the two soil layers responded differently to the addition of amendments. Where an OTU was higher in abundance in the topsoil, there was a tendency for that OTU to be more abundant in the fertilizer and then poultry litter treatments compared to the nil-amendment control. Similarly, where an OTU was higher in abundance in the subsoil, the poultry litter and then nil treatments tended to be higher in abundance than the synthetic fertilizer. The addition of nutrients or organic matter has been shown to stimulate microbial activity and alter the abundance of select species^10,20,21^. It appears that poultry litter had a stimulatory effect on selected microbes in both soil layers, but the fertilizer had a stimulatory effect in the topsoil and an inhibitory effect in the subsoil. This could be due to the chemical composition of the amendments, since differing C chemistry and nutrient content is known to affect microbial communities^29,30^. The inorganic fertilizer contained highly available macro- and micronutrients whereas the poultry litter contained both nutrients and carbon. Nutrients from the fertilizer might have leached down the soil profile, resulting in an increase of solutes in the soil solution that might have negatively affected the microbial communities there.

Soil and plant properties played a role in shaping microbial community structure in this experiment, with plant roots and soil structure being identified as key drivers. In contrast, soil inorganic N concentration and aboveground plant variables were not strongly correlated with either bacterial or fungal community structure. The structure and function of soil microbial communities are known to be strongly linked to the physical soil environment and to plant roots, with complex interactions occurring between soil biota, roots and aggregates^31,32^. However, much of the variation in bacterial and fungal community structure in this experiment remained unexplained. It is likely that other edaphic variables – such as pH and soil carbon^33,34^ – may also have been key drivers of microbial community structure after amendment but these were not analyzed in the present study.

Microbial diversity was found to be negatively correlated with soil chemical fertility and plant growth in this experimental system. The richness, diversity and evenness of the bacterial and fungal communities almost always decreased as N concentrations in the soil and shoot, and shoot and root biomass, increased. These results suggest that microbial diversity was not necessarily a critical determinant of increased plant biomass and yield in the present study, since alpha diversity indices were not positively correlated with measures of plant productivity. Many authors have reported strong positive relationships between measures of microbial diversity, soil fertility and plant productivity, indicating that diversity is critical to maintaining ecosystem services^35–38^. However, others contend that this relationship is complex and should not be generalized^39^ and that changes in community composition and activity, rather than diversity, affect ecosystem processes^21,40^. This experiment demonstrates that a loss of species or reduction in the diversity and evenness of the microbial community does not always have a negative impact on plant growth or yield. Increased root growth and exudation may actually have been a driver of reduced diversity in the soil due to plant selection pressures on soil biota^41,42^. Additionally, variation in plant growth and microbial communities could be attributed to differences in carbon and nutrient availability of the different amendments, since we suspect that this might have affected microbial community structure and diversity. Because nutrients have a first-order control on both plant productivity and soil microbial communities, manipulating nutrients as we have done here could result in both direct and indirect effects on microbial community structure and diversity, complicating our interpretation of the plant-microbial relationship. The contribution of microbes to plant productivity, and the diversity of the microbial community, is suggested to decline with increasing nutrient availability in a system^7^. Other authors have suggested a decoupling of microbial diversity-plant productivity relationships in degraded ecosystems due to changes in soil nutrient condition and physical structure altering microbial community composition, abundance and diversity^43^ These degraded ecosystems may have some parallels to the heavily-disturbed experimental units used in this experiment, albeit they occur at vastly different temporal and spatial scales.

Finally, there were no obvious large-scale shifts in the functional potential of the microbial community arising from the different amendments applied and their interaction with placement depth. It was expected that the bacterial and fungal OTUs responding to the experimental treatments would have functional roles related to nutrient cycling, carbon decomposition, plant growth promotion and so on^7^. Instead, extrapolating from the OTUs that were observed to differ in abundance between treatments, we observed disparate changes in function that did not support this theory. A small minority of bacterial and fungal OTUs was observed to differ in abundance based on amendment type or placement depth. Many of these OTUs belonged to phyla we know little about, such as Candidatus Saccharibacteria (Candidate division TM7) or unclassified Acidobacteria taxa. Others we can make functional predictions for, such as the Ascomycota which are known to influence soil structure via their hyphae or the N-fixing Xanthobacteraceae, but these observations are not consistent and do not point to a large-scale shift in function after addition of amendment. Further investigation of shifts in microbial community function after addition of amendments requires a more direct approach utilizing techniques such as qPCR or omics-based tools to target functional changes more directly^44,45^.

## Conclusion

This experiment revealed that the addition of poultry litter and inorganic fertilizer significantly altered microbial community structure and diversity in the topsoil and subsoil of repacked soil columns. The amendments changed the composition of bacterial and fungal communities by stimulating or suppressing taxa, leading to divergence of the amended soils. Substantial inherent heterogeneity in microbial communities in topsoil and subsoil layers was also observed. However, microbial diversity was found to be negatively correlated with plant productivity in this experimental system. We hypothesize that these findings could be due to the chemical composition of the amendments and the differing release rates of nutrients, which affected both the microbes and the plant.

## Methods

### Treatments and experimental design

This controlled environment experiment with wheat plants grown in repacked soil columns used a factorial design with 3 amendments (nil-amendment control, poultry litter, slow-release synthetic fertilizer) × 2 depths of placement (topsoil, subsoil) treatments. There were three replicates of each treatment for a total of six treatments: (1) topsoil + nil-amendment control (‘TOP NIL’), (2) topsoil + poultry litter (‘TOP PL’), (3) topsoil + fertilizer (‘TOP FERT’), (4) subsoil + nil-amendment control (‘SUB NIL’), (5) subsoil + poultry litter (‘SUB PL’) and (6) subsoil + fertilizer (‘SUB FERT’). There were two complete sets of six treatments to allow destructive harvest at two time points. All columns were planted to spring wheat (*Triticum aestivium* cv. Gauntlet). The addition of ± plant treatments was not possible due to experimental constraints.

The soil used was a Solonetz^46^ that was collected from a property in Ballan in south-eastern Australia. Topsoil (0–15 cm) and subsoil (20–40 cm) were collected and then all soil was air-dried, crushed and sieved to 2 mm. The topsoil had pH(1:5 CaCl_2_) 4.7, organic C 38 g kg^−1^, total N 4.1 g kg^−1^, Olsen P 34 mg kg^−1^, electrical conductivity (EC, 1:5 water) 0.39 dS m^−1^, cation exchange capacity (CEC) 8.3 cmol kg^−1^ and the texture was 57% sand, 23% silt and 21% clay. The subsoil had pH(CaCl_2_) 4.9, organic C 8.6 g kg^−1^, total N 1.2 g kg^−1^, Olsen P 5.8 mg kg^−1^, EC 0.06 dS m^−1^, CEC 4.2 cmol kg^−1^ and the texture was 53% sand, 26% silt and 21% clay.

The poultry litter was sourced from a broiler operation and sieved through a 4-mm mesh screen. Poultry litter was applied at a rate of 53.3 g per column (equivalent to 30 t ha^−1^). The slow-release synthetic fertilizer Macracote Orange (Langley Fertilizers; Perth, Australia) was applied at 15 g per column to achieve a similar total N as the poultry litter equivalent to 1,200 kg N ha^−1^. The poultry litter contained (% w/w): 34 C, 4.5 N, 1.7 P, 2.7 K and 0.68S. The slow-release synthetic fertilizer contained (% w/w) 16 N (7.8 as CH_4_N_2_O, 4.8 as NH_4_^+^ and 3.4 as NO_3_^−^), 3.5 P (3.2 water-soluble P and 0.3 citrate-soluble P), 10 K and 5.2S (as K_2_SO_4_).

### Experimental unit construction

**Figure 5:**
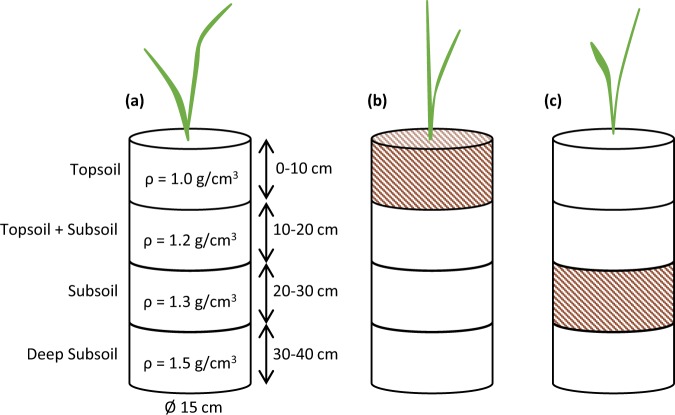
Diagram of the different soil layers and amendment placement locations in nil-amendment control **(a)**, topsoil amendment **(b)** and subsoil amendment **(c)** experimental columns.

PVC columns of 15 cm in diameter and 40 cm tall were filled with 8.7 kg soil in four layers to simulate the texture-contrast profile of the Solonetz soil. The layers were constructed as follows: 0–10 cm of topsoil, 10–20 cm of a 1:1 topsoil:subsoil blend, 20–30 cm of subsoil and another 30–40 cm of subsoil (Fig. 5). The column was tapped to compact the soil to the desired bulk density of 1.5 g cm^−3^ in the deep subsoil. The poultry litter and slow-release synthetic fertilizer were thoroughly mixed into either the topsoil (Fig. 5a) or subsoil (Fig. 5b) layer.

Basal nutrients were mixed into the 0–10 cm layer of each column at the following rates (mg kg^−1^ soil): 180 KH_2_PO_4_; 120 K_2_SO_4_; 180 CaCl_2_.2H_2_O; 50 MgSO_4_.7H_2_O; 15 MnSO_4_.H_2_O; 9 ZnSO_4_.7H_2_O; 6 CuSO_4_.5H_2_O; 0.4 Na_2_MoO_4_.2H_2_O; 5.5 FeEDTA. A watering pipe was fitted in the center of each column to allow watering of deeper soil layers and moisture probes were inserted into the top of the 10–20 cm and/or 30–40 cm layers of some of the columns to monitor soil moisture throughout the experiment. The soil surface was covered by 2 cm of high-density polyethylene beads to minimize evaporation and a thin layer of beads was added at each 10 cm layer to distinguish them at harvest.

### Growing conditions

The experiment was carried out in a controlled environment room with a 14-hour photoperiod, day-time temperature of 22.5 °C and night temperature of 18.5 °C. The irradiance increased from 350 to 650 μmol m^2^ s^−1^ at the canopy level as plant height increased. All columns in the controlled environment room had been grouped into three blocks (three shelves) with re-randomization within each shelf twice a week. The room had an atmospheric CO_2_ concentration of 480–500 ppm and a relative humidity of 56%.

Twelve pre-germinated seeds of wheat of similar size were sown in each column on Day 0. These were thinned to five plants per column 13 days after sowing (DAS) and two plants per column 30 DAS. The young shoots were cut at the soil surface, leaving their roots to decompose in the soil. Urea was applied to all columns at a rate of 30 mg N kg^−1^ soil 37 DAS to alleviate possible N deficiency in organic amendment treatments.

All columns were watered daily to approximately 80% of field capacity. The topsoil was watered from the surface and watering tubes were used to wet the subsoil layers.

### Sampling and measurement

The experimental units were destructively harvested. One set of columns was harvested at 76 DAS (hard dough to early ripening, Zadoks 87–91^47^) and the second set at 107 DAS (physiological maturity, Zadoks 99^47^). Shoots were cut at the soil surface and dried at 70 °C for 72 hours before recording the mass. For the second harvest, all ears were removed and threshed to separate the grain kernels before the total grain weight was recorded.

Detailed measurements were conducted for the samples of the first harvest at 76 DAS. A subsample of shoot biomass was ground with a ball mill and N concentration was measured with a Series II CHNS/O Analyzer (PerkinElmer; Colorado, USA). Soil from each column was sectioned into the four 10-cm layers and a subsample was collected for analysis of the soil microbial community, gravimetric moisture content, inorganic N and water-stable aggregates. The rest of the soil from each layer was sieved and washed to retrieve the roots. Subsamples of roots were scanned and analyzed with WinRHIZO (Regent Instruments Inc.; Canada) for root length, surface area and diameter. Roots were then dried at 70 °C for 72 hours before recording the mass.

Soil inorganic N was measured with a QuickChem® 8500 Series II FIA Automated Ion Analyzer (Lachat Instruments; USA) after extraction with 2 M KCl (1:5 soil to solution ratio), shaking for 1 hour and filtering with No. 1 Whatman filter paper. Aggregate size distribution was determined using a standard wet-sieving apparatus whereby 25 g of 10 mm-sieved, air-dried soil was placed on top of a nest of pre-weighed sieves and immersed in deionized water for 10 min. The stack of sieves was then subjected to automatic vertical movement for 15 min at 70 rpm. Four aggregate size fractions were collected: large macroaggregates (>2 mm), small macroaggregates (0.25–2 mm), microaggregates (0.053–0.25 mm) and the silt and clay fraction (<0.053 mm). Mean-weight diameter (MWD) of soil aggregates was calculated using the standard formula:

$${\rm{MWD}}=\sum {x}_{i}{w}_{i}$$

where *x*_i_ is the mean aperture of the adjacent sieves and *w*_i_ is the mass fraction remaining on each sieve^48^.

Genomic DNA was extracted from 0.25 g soil samples using the Mobio PowerSoil DNA Isolation Kit as per the manufacturer’s instructions. DNA concentration and purity were determined using an Implen P330 NanoPhotometer (Implen GmbH, Munich, Germany) and Qubit 3.0 Fluorometer (ThermoFisher Scientific; Invitrogen, MA, USA). 16S rRNA and ITS diversity profiling was performed by the Australian Genome Research Facility (Melbourne, Australia) on an Illumina MiSeq (Illumina Inc., CA, USA) platform. A 300 bp target was amplified from the V3-V4 region of the 16S rRNA gene using primers 341 F (5′- CCTAYGGGRBGCASCAG) and 806 R (5′- GGACTACNNGGGTATCTAAT)^49,50^ and an approximately 230 bp target was amplified from the ITS1-ITS2 region of the internal transcribed spacer (ITS) using primers ITS1f (5′- CTTGGTCATTTAGAGGAAGTAA) and ITS2 (5′- GCTGCGTTCTTCATCGATGC) (White *et al*. 1990, Gardes and Bruns 1993).

### Bioinformatics

Raw, demultiplexed fastq files from the Australian Genome Research Facility were re-barcoded, joined and quality filtered using the UPARSE pipeline^51^. Joined paired-end reads were quality-filtered by discarding reads with total expected errors > 1 and removing singletons. Operational taxonomic units (OTUs) were clustered with a minimum cluster size > 2 and 97% similarity cut off to enable detection of community-level changes using the UPARSE clustering algorithm. Taxonomic assignments were performed using the USEARCH UTAX algorithm with reference databases created using the RDP 16S (version 16) and UNITE ITS (version 7) training datasets (available at https://www.drive5.com/usearch/). The minimum percentage identity required for an OTU to consider a database match a hit was 80%. OTUs identified as chloroplasts and mitochondrial DNA were removed from the data set and all OTUs with lower than 80% taxonomic confidence threshold were denoted as ‘Unassigned.’ A phylogenetic tree was constructed using the UPGMA algorithm in MUSCLE^52^.

### Statistical analysis

All analyses were carried out using R version 3.5.0^53^ and PRIMER version 7 software with the PERMANOVA + add-on^54,55^. Plots were produced in R with the assistance of packages *ggplot2*^56^ and *RColorBrewer*^57^. Using package *phyloseq*^58^, spurious reads were removed using a 0.005% relative abundance cut-off^59^ and the bacterial and fungal data sets were rarefied to 17,000 and 3,500 reads, respectively (Supplementary Fig. S1). Alpha diversity analyses (Chao1 richness estimator^17^, Shannon diversity index (*H*)^18,19^) were performed on bacterial and fungal OTU tables using the *estimate_richness* function in the package *phyloseq*^58^. Simpson’s evenness (*E*) was calculated by dividing the inverse Simpson’s index by observed species richness^19^. Weighted and unweighted UniFrac distances between samples^60^ were calculated in *phyloseq*^58^. Principal coordinates analysis of weighted UniFrac distances was used to visualize the relationships and differences between treatments. Global and pairwise permutational multivariate analysis of variance (PERMANOVA) on the distance matrices was performed in using the PERMANOVA + add-on in PRIMER^54^. All PERMANOVA tests used 9999 permutations from unrestricted permutation of raw data. Where there were fewer than 99 unique permutations for a meaningful test, approximate Monte Carlo *P*-values (*P*(MC)) were obtained from an asymptotic permutation distribution^55^. Package *mvabund*^61^ was used to determine which microbial OTUs differed significantly (*P*_adj_ < 0.05) in abundance between amendment and depth treatments. For this procedure, unrarefied sequence counts were modelled on negative binomial distributions in the generalized linear models. Pearson’s correlation analysis was performed using the *rcorr* function in package *Hmisc*^62^ to determine relationships between alpha diversity indices and environmental variables. Function *oridstep* in package *vegan*^63^ was used to select significant (*P* < 0.05) drivers of microbial community composition from a standardized matrix of the 15 environmental variables (Supplementary Table S4) using both forward and backward selection. Highly correlated (R^2^ > 0.90) variables were removed prior to model selection. Canonical correspondence analysis (CCA) was carried out using *vegan*^63^ to correlate sample ordination with the retained variables. Variation partitioning analysis was used to quantify the effects of the significantly correlated environmental variables on the microbial community composition using *varpart* function in *vegan*^63^.

## Supplementary information

Dataset 1

## Data Availability

The datasets generated during the current study are available from the corresponding author on reasonable request.

## References

[1] Diacono M, Montemurro F (2010). Long-term effects of organic amendments on soil fertility. A review. Agronomy for Sustainable Development.

[2] Celestina C (2018). Crop yield responses to surface and subsoil applications of poultry litter and inorganic fertiliser in south-eastern Australia. Crop & Pasture Science.

[3] Khalilian A, Williamson RE, Sullivan MJ, Mueller JD, Wolak FJ (2002). Injected and broadcast application of composted municipal solid waste in cotton. Applied Engineering in Agriculture.

[4] Leskiw LA, Welsh CM, Zeleke TB (2012). Effect of subsoiling and injection of pelletized organic matter on soil quality and productivity. Canadian Journal of Soil Science.

[5] Gill JS, Sale PWG, Tang C (2008). Amelioration of dense sodic subsoil using organic amendments increases wheat yield more than using gypsum in a high rainfall zone of southern Australia. Field Crops Research.

[6] Gill JS, Sale PWG, Peries R, Tang C (2009). Changes in soil physical properties and crop root growth in dense sodic subsoil following incorporation of organic amendments. Field Crops Research.

[7] van der Heijden MGA, Bardgett RD, van Straalen NM (2008). The unseen majority: Soil microbes as drivers of plant diversity and productivity in terrestrial ecosystems. Ecology Letters.

[8] Clark GJ, Dodgshun N, Sale PWG, Tang C (2007). Changes in chemical and biological properties of a sodic clay subsoil with addition of organic amendments. Soil Biology & Biochemistry.

[9] Clark GJ, Sale PWG, Tang C (2009). Organic amendments initiate the formation and stabilisation of macroaggregates in a high clay sodic soil. Australian Journal of Soil Research.

[10] Das S, Jeong ST, Das S, Kim PJ (2017). Composted cattle manure increases microbial activity and soil fertility more than composted swine manure in a submerged rice paddy. Frontiers in Microbiology.

[11] Blanchet G, Gavazov K, Bragazza L, Sinaj S (2016). Responses of soil properties and crop yields to different inorganic and organic amendments in a Swiss conventional farming system. Agriculture, Ecosystems and Environment.

[12] Dawe D (2003). Do organic amendments improve yield trends and profitability in intensive rice systems?. Field Crops Research.

[13] Edmeades DC (2003). The long-term effects of manures and fertilisers on soil productivity and quality: a review. Nutrient Cycling in Agroecosystems.

[14] Chen Y, Camps-Arbestain M, Shen Q, Singh B, Cayuela ML (2018). The long-term role of organic amendments in building soil nutrient fertility: a meta-analysis and review. Nutrient Cycling in Agroecosystems.

[15] Hijbeek R (2017). Do organic inputs matter – a meta-analysis of additional yield effects for arable crops in Europe. Plant and Soil.

[16] Geisseler D, Scow KM (2014). Long-term effects of mineral fertilizers on soil microorganisms - A review. *Soil Biology &*. Biochemistry.

[17] Chao A (1984). Nonparametric estimation of the number of classes in a population. Scandinavian Journal of Statistics.

[18] Shannon Claude E., Weaver Warren, Wiener Norbert (1950). The Mathematical Theory of Communication. Physics Today.

[19] Magurran, A. E. *Measuring Biological Diversity*, 10.1017/CBO9781107415324.004 (Blackwell Publishing, 2004).

[20] Banerjee S (2016). Network analysis reveals functional redundancy and keystone taxa amongst bacterial and fungal communities during organic matter decomposition in an arable soil. *Soil Biology &*. Biochemistry.

[21] Bastida F, Selevsek N, Torres IF, Hernández T, García C (2015). Soil restoration with organic amendments: linking cellular functionality and ecosystem processes. Nature Scientific Reports.

[22] Staley C (2018). Urea amendment decreases microbial diversity and selects for specific nitrifying strains in eight contrasting agricultural soils. Frontiers in Microbiology.

[23] Flavel TC, Murphy DV (2006). Carbon and nitrogen mineralization rates after application of organic amendments to soil. Journal of Environmental Quality.

[24] Borda-Molina D, Seifert J, Camarinha-Silva A (2018). Current perspectives of the chicken gastrointestinal tract and its microbiome. Computational and Structural Biotechnology Journal.

[25] Kumar U (2018). Continuous application of inorganic and organic fertilizers over 47 years in paddy soil alters the bacterial community structure and its influence on rice production. Agriculture, Ecosystems and Environment.

[26] Chen C (2016). Microbial communities of an arable soil treated for 8 years with organic and inorganic fertilizers. Biology and Fertility of Soils.

[27] Sun R (2016). Fungal community composition in soils subjected to long-term chemical fertilization is most influenced by the type of organic matter. Environmental Microbiology.

[28] Sanaullah M (2016). How do microbial communities in top- and subsoil respond to root litter addition under field conditions? *Soil Biology &*. Biochemistry.

[29] Cozzolino V, Di Meo V, Monda H, Spaccini R, Piccolo A (2015). The molecular characteristics of compost affect plant growth, arbuscular mycorrhizal fungi, and soil microbial community composition. Biology and Fertility of Soils.

[30] Baumann K, Marschner P, Smernik RJ, Baldock JA (2009). Residue chemistry and microbial community structure during decomposition of eucalypt, wheat and vetch residues. Soil Biology & Biochemistry.

[31] Tecon R, Or D (2017). Biophysical processes supporting the diversity of microbial life in soil. FEMS Microbiology Reviews.

[32] Feeney DS (2006). Three-dimensional microorganization of the soil-root-microbe system. Microbial Ecology.

[33] Delgado-Baquerizo M (2017). It is elemental: soil nutrient stoichiometry drives bacterial diversity. Environmental Microbiology.

[34] Fierer N, Jackson RB (2006). The diversity and biogeography of soil bacterial communities. Proceedings of the National Academy of Sciences.

[35] Delgado-Baquerizo M (2017). Circular linkages between soil biodiversity, fertility and plant productivity are limited to topsoil at the continental scale. New Phytologist.

[36] Delgado-Baquerizo M (2016). Microbial diversity drives multifunctionality in terrestrial ecosystems. Nature Communications.

[37] Fanin N (2018). Consistent effects of biodiversity loss on multifunctionality across contrasting ecosystems. *Nature Ecology &*. Evolution.

[38] Maron P-A (2018). High microbial diversity promotes soil ecosystem functioning. Applied and Environmental Microbiology.

[39] Jung J, Philippot L, Park W (2016). Metagenomic and functional analyses of the consequences of reduction of bacterial diversity on soil functions and bioremediation in diesel-contaminated microcosms. Nature Scientific Reports.

[40] Nielsen UN, Ayres E, Wall DH, Bardgett RD (2011). Soil biodiversity and carbon cycling: A review and synthesis of studies examining diversity-function relationships. European Journal of Soil Science.

[41] Hartmann A, Schmid M, van Tuinen D, Berg G (2009). Plant-driven selection of microbes. Plant and Soil.

[42] Corneo PE, Suenaga H, Kertesz MA, Dijkstra FA (2016). Effect of twenty four wheat genotypes on soil biochemical and microbial properties. Plant and Soil.

[43] Li Y (2016). Changes of soil microbial community under different degraded gradients of alpine meadow. Agriculture, Ecosystems and Environment.

[44] Jansson JK, Baker ES (2016). A multi-omic future for microbiome studies. Nature Microbiology.

[45] Desai C, Pathak H, Madamwar D (2010). Advances in molecular and ‘-omics’ technologies to gauge microbial communities and bioremediation at xenobiotic/anthropogen contaminated sites. Bioresource Technology.

[46] IUSS Working Group WRB. *World Reference Base for Soil Resources 2014, Update 2015. International Soil Classification System for Naming Soils and Creating Legends for Soil Maps. World Soil Resources Reports No. 106*. (FAO, 2015).

[47] Zadoks JC, Chang TT, Konzak CF (1974). A decimal code for the growth stages of cereals. Weed Research.

[48] van Bavel CHM (1950). Mean weight-diameter of soil aggregates as a statistical index of aggregation. Soil Science Society of America Journal.

[49] Yu Y, Lee C, Kim J, Hwang S (2005). Group-specific primer and probe sets to detect methanogenic communities using quantitative real-time polymerase chain reaction. Biotechnology and Bioengineering.

[50] Gardes M, Bruns TD (1993). ITS primers with enhanced specificity for basidiomycetes - application to the identifcation of mycorrhizae and rusts. Molecular Ecology.

[51] Edgar RC (2013). UPARSE: highly accurate OTU sequences from microbial amplicon reads. Nature Methods.

[52] Edgar RC (2004). MUSCLE: Multiple sequence alignment with high accuracy and high throughput. Nucleic Acids Research.

[53] R Core Team. R: A Language and Environment for Statistical Computing. R Foundation for Statistical Computing, Vienna, Austria. (At: https://www.R-project.org/) (2018).

[54] Clarke, V. R. & Gorley, R. N. PRIMER v7: User Manual/Tutorial. PRIMER-E, Plymouth (2015).

[55] Anderson, M., Gorley, R. N. & Clarke, K. R. PERMANOVA+ for PRIMER: Guide to Software and Statistical Methods. PRIMER-E, Plymouth (2008).

[56] Wickham, H. ggplot2: Elegant Graphics for Data Analysis. R package version 2.2-1. (At: https://cran.r-project.org/package=ggplot2/) (2018).

[57] Neuwirth, E. RColorBrewer: ColorBrewer Palettes. R package version 1.1-2. (At: https://CRAN.R-project.org/package=RColorBrewer/) (2014).

[58] McMurdie PJ, Holmes S (2013). phyloseq: An R package for reproducible interactive analysis and graphics of microbiome census data. PLoS ONE.

[59] Bokulich NA (2013). Quality-filtering vastly improves diversity estimates from Illumina amplicon sequencing. Nature Methods.

[60] Lozupone C, Knight R (2005). UniFrac: a new phylogenetic method for comparing microbial vommunities. Applied and Environmental Microbiology.

[61] Wang Y, Naumann U, Wright ST, Warton D (2012). I. mvabund – an R package for model-based analysis of multivariate abundance data. Methods in Ecology and Evolution.

[62] Harrell, F. E. Hmisc: Harrell Miscellaneous. R package version 4.1-1. (At: https://CRAN.R-project.org/package=Hmisc/) (2018).

[63] Oksanen, J. *et al*. vegan: Community Ecology Package. R package version 2.4-6. (At: https://CRAN.R-project.org/package=vegan/) (2018).

